# Etymologia: Sunda Pangolin

**DOI:** 10.3201/eid2707.ET2707

**Published:** 2021-07

**Authors:** Clyde Partin

**Affiliations:** Emory University School of Medicine, Atlanta, Georgia, USA

**Keywords:** Sunda pangolin, Manis javanica, pangolin, spiny anteater, scales, intermediate host, coronavirus disease, COVID-19, pandemic, endangered species, Malaysia, Indonesia

## Sunda Pangolin [′sün də ′paNG ɡōl ən]

The Sunda or Malayan pangolin (*Manis javanica*) achieved notoriety during the Coronavirus disease pandemic because of flawed evidence suggesting that pangolins could be intermediate hosts (Figure). Genetic analysis later demonstrated that the spike protein angiotensin-converting enzyme-2 receptor-binding domain of the pangolin had marginal viral avidity and thus was an unlikely infectious conduit. Pangolins are edentate mammals possessing short powerful forelimbs suitable for excavating ants and termites.

**Figure:**
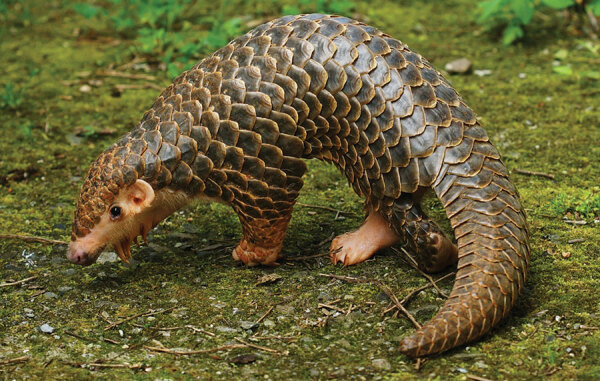
Covered in tough keratin scales interspersed with strands of fur, the pangolin, also known as a scaly anteater, assumes an impenetrable rolled-up position when threatened. Note the short muscular forelimbs. Pangolins are endangered and World Pangolin Day is the third Saturday in February. Photo of a young Chinese pangolin (*Manis pentadactyla*) by Te-Chuan Chan (Taipei Zoo, Taiwan) and Wen-Ta Li (Pangolin International Biomedical Consultant Ltd., Taiwan)

Linnaeus named the genus *Manis*, derived from *manes*, Latin for “spirits” or “ghosts or shades of the dead,” which refers to their noncuddly reptilian persona and solitary nocturnal foraging. Covered by keratin scales, pangolins, when threatened, assume a rolled up position, described by the Malayan word pengguling (one who rolls up). Native to Java (thus *javanica*), their habitat includes Southeast Asia, especially the Indomalayan archipelago and Sunda Islands. Humans hunt pangolins for their meat, consume their blood as an elixir, and use their scales and other body parts as ingredients for crafting leather products and nonefficacious medications. 
